# Multi-Year Analysis of Demographic Reporting in Ophthalmology Artificial Intelligence Studies

**DOI:** 10.3390/vision10040072

**Published:** 2026-09-25

**Authors:** Joshua Ong, Patrice M. Hicks, Yazan K. Felwa, Jenna N. Hart, Ming-Chen Lu, Travis K. Redd, Kate M. Saylor, Sophia Y. Wang, Maria A. Woodward

**Affiliations:** 1 Department of Ophthalmology and Visual Sciences, University of Michigan, Ann Arbor, MI 48105, USA; jong8@meei.harvard.edu (J.O.); pmhicks@med.umich.edu (P.M.H.); yfelwa@umich.edu (Y.K.F.); mingchlu@med.umich.edu (M.-C.L.); 2 Department of Ophthalmology, Harvard Medical School, Massachusetts Eye and Ear, Boston, MA 02114, USA; 3 School of Medicine, Wayne State University, Detroit, MI 48201, USA; cp8564@wayne.edu; 4 Department of Ophthalmology, University of Colorado Anschutz, Aurora, CO 80045, USA; travis.redd@cuanschutz.edu; 5 Taubman Health Sciences Library, University of Michigan, Ann Arbor, MI 48109, USA; kmacdoug@med.umich.edu; 6 Byers Eye Institute, Stanford University, Palo Alto, CA 94303, USA; sywang@stanford.edu; 7 Institute for Healthcare Policy and Innovation, University of Michigan, Ann Arbor, MI 48109, USA

**Keywords:** artificial intelligence, demographic, ethnicity, gender, ophthalmology, sex, race

## Abstract

Sex, gender, race, ancestry, and ethnicity data are critical to ensuring an equitable, generalizable artificial intelligence (AI) framework for clinical applications in ophthalmology. The primary question is whether reporting demographic data in ophthalmology AI studies can be improved. This retrospective study investigated AI studies between June 2020 and June 2023. Reporting of sex, gender, race, and ethnicity data was stratified by publication year and institution. A sub-analysis of National Institutes of Health (NIH)-funded manuscripts assessed adherence to the 2020–2021 updated guidance for data reporting in science and medical journals. Adherence was assessed based on terminology used in the published manuscript; it does not indicate that the underlying construct or assessment method was verified. In total, 1005 articles were screened across 15 journals. Of the 268 included studies, 122 studies reported sex demographics, 35 studies reported gender demographics, and 96 studies reported race, ethnicity, or ancestry demographics. In 2020, 46% of studies reported either race, ethnicity, or ancestry. This frequency decreased from 46% in 2020 to 24% in 2021, followed by a modest increase in 2022 to 44%, and a slight decrease in 2023 to 35%. Sex and gender were reported for 53.57% and 10.71% of studies in 2020, 40% and 6% in 2021, 50% and 17% in 2022, and 44% and 17% in 2023, respectively. These results highlight gaps in demographic reporting. To ensure fair clinical tools, AI ophthalmic research can improve data transparency. Academic journals may require the reporting of demographic data per NIH guidelines to better understand AI tool generalizability.

## 1. Introduction

Artificial intelligence (AI) in ophthalmology has emerged as a promising technology to enhance ophthalmic care [1]. AI systems in ophthalmology, including machine learning and deep learning techniques, have been shown to be highly accurate in detecting various diseases such as diabetic retinopathy, age-related macular degeneration, and glaucoma [2]. These advances present an exciting future for earlier detection of ophthalmic diseases, with the goal of prolonging and protecting patient vision.

While the deployment of AI in ophthalmology is an exciting prospect, the equitable integration of AI requires a thorough examination of how datasets and AI systems are constructed to include diverse demographic groups. There is a critical need for transparent reporting of demographic information to ensure that AI technologies improve ophthalmic care for all, rather than widen disparities. Biased AI tools have been shown to perpetuate or exacerbate existing health disparities [3,4]. Ethical real-world implementation requires the technology to be generalizable across population groups during the development and validation phases of AI tools [5]. Various ophthalmic conditions often manifest with distinct characteristics in different patient populations, which can degrade AI performance when models are applied to other populations [6,7,8].

As AI applications in ophthalmology rapidly expand, ensuring that these tools are trustworthy, generalizable, and clinically equitable has become increasingly important [9]. Model performance may vary across patient populations; thus, transparent reporting of demographic characteristics is essential for assessing fairness, identifying potential bias, and assessing whether study findings are applicable to diverse clinical settings [10]. Inadequate representation and reporting may further exacerbate existing health disparities and undermine accountability in AI-assisted patient care [11]. However, the extent to which demographic characteristics are reported in ophthalmic AI research remains unclear. Therefore, we investigated the reporting of race, ethnicity, sex, and gender in AI studies in ophthalmology to characterize current limitations and identify opportunities to improve demographic reporting.

## 2. Materials and Methods

Institutional review board approval was not required as this study did not involve human participants per institutional guidelines.

### 2.1. Study Selection and Screening

Original studies investigating the use of AI in ophthalmology between 1 June 2020 and 30 June 2023 were identified from PubMed because the search was limited to 15 journals. We included studies published in the 15 ophthalmology journals most cited in the Clarivate Science Citation Index Expanded database. This date range was chosen because of the recent focus on correct classification of demographic characteristics and updated guidance on reporting in medical journals, science journals, and by the National Institutes of Health (NIH), following the start of the COVID-19 pandemic and prominent cases of race-related violence [12,13,14]. These events prompted sectors to critically examine and address their roles in perpetuating inequality. Studies were identified by search terms and filters including “artificial intelligence, natural language processing, machine learning, artificial neural network, and deep learning” and others as fully described in Online Supplemental Figure S1. Articles were screened in three rounds by three reviewers (Y.K.F., P.M.H., J.N.H.) using Covidence (Veritas Health Innovation, Melbourne, Victoria, Australia). First, article titles and abstracts were reviewed by at least two reviewers. Next, full texts were reviewed by at least two reviewers. When discrepancies occurred, a third reviewer served as the adjudicator. Lastly, articles that included development of AI in ophthalmology using participant data were extracted. During data extraction, reviewers underwent training using a random sample of 15 studies; conflicts were reviewed as a group before proceeding with a standardized review process. Three reviewers completed the remaining data extraction independently (Y.K.F., P.M.H., J.N.H.). Screening was conducted in Covidence (Veritas Health Innovation, Melbourne, Australia).

### 2.2. Data Extraction Process

Data were extracted from the version of the article available on the publishers’ websites. The extracted data included study characteristics (publication title, year of publication, authors, journal, the location of the principal investigator’s institution [United States (US) or international], and diagnosis or condition studied), demographic information (sex, gender, race, ethnicity, ancestry, and method of demographic collection), and limitations mentioned by authors. Demographic reporting of sex, gender, race, and ethnicity was chosen because NIH has implemented policies requiring the collection and transparent reporting of sex, women, and minority data in NIH-funded studies [15,16]. The definitions and associated categorizations for the following demographic variables were used: sex is a biological concept rooted in physiology, anatomy, hormones, and genetics, with typical categories including male, female, and intersex [17]. Gender is a multifaceted concept that includes both gender expression and identity; examples include woman, man, transgender, and non-binary/non-conforming [18]. Race is a socially constructed concept used to categorize individuals [19]. The five minimum categories of race include Black or African American, White or Caucasian, Asian, Native Hawaiian or Other Pacific Islander, and American Indian or Alaska Native [20]. Ethnicity is defined by the social and cultural traits shared by a group of people, including their language, religion, beliefs, values, and behaviors, with common categories including non-Hispanic and Hispanic [21].

Studies were considered compliant for each specific data category (sex, gender, race, ethnicity, or ancestry) if they stated that they collected data on that specific variable. Studies were recorded as following guidelines if they collected the demographic data and utilized the NIH guideline terminology at the time of data extraction [17,18,19,20,21,22]. For example, a study was considered compliant with the guidelines if it reported collecting sex data and provided percentages of participants identified as male and female. In contrast, if a study collected sex data but reported percentages of participants who were women or men, it was considered non-compliant with the guidelines. This was repeated for gender, race, and ethnicity data collection based on NIH guidelines for terminology at the time of the data extraction for all studies. Manuscripts were marked as adequately mentioning the limitation of not assessing race, ethnicity, sex, or gender if the discussion section noted that these demographic factors were not assessed as a limitation, or if it indicated that future research should assess differences related to these demographic factors or the need to conduct the study in additional racial, ethnic, sex, or gender groups. For example, one study met the criteria as it stated, “patient ethnicity was not provided in our data set’s metadata.”

### 2.3. Data Analysis

We conducted a global analysis including all manuscripts, and two sub-analyses of manuscripts: (1) that assessed only studies that had NIH funding, and (2) that assessed any study that included US data, including studies with data from both the US and another country. This methodology was chosen because race and ethnicity are viewed differently in the US than internationally. For the global analysis, we grouped race, ethnicity, and ancestry to assess the reporting status. For the NIH-funded and US sub-analyses, we assessed race and ethnicity reporting separately using guidelines set by the NIH at the time of data extraction [17,18,19,20,21,22]. The NIH guidelines specify standards for racial and ethnic categories, noting that these are sociopolitical constructs rather than anthropological classifications.

Study characteristics and demographic information were summarized with frequencies and percentages (%). Differences in demographic reporting status between study years were compared using Fisher’s exact test. Statistically significant *p*-values were followed by post hoc Holm-adjusted pairwise comparisons. A *p-value* ≤0.05 was considered statistically significant. All statistical analyses were performed using R version 4.3.2 (including the rstatix package, R Foundation for Statistical Computing, Vienna, Austria).

## 3. Results

The domains investigated in this study included the year of publication; whether the manuscript reported sex or gender and race, ethnicity, or ancestry; and whether it identified limitations related to these characteristics. For the sub-analyses of NIH-funded and U.S.-based studies, we examined the year of publication; whether the manuscript reported sex, gender, race, or ethnicity; whether the manuscript followed NIH reporting guidelines at the time of data extraction; and how these characteristics were assessed. We also examined whether manuscripts acknowledged any limitations related to sex, gender, race, or ethnicity. In total, 1005 records were screened, 376 full-text articles were assessed for eligibility, and 268 studies met the inclusion criteria and were included in the analysis (Figure 1).

### 3.1. Overview of All Studies Included in the Analysis

Among the 268 studies identified between June 2020 and June 2023 in PubMed, the three journals with the most AI studies included were: British Journal of Ophthalmology (*n* = 63, 23.51%), American Journal of Ophthalmology (*n* = 60, 22.39%), and Ophthalmology (*n* = 26, 9.70). The full list of journals and numbers of studies extracted are listed in Table 1. Most studies were published in 2022 (32.09%), followed by 2021 (30.97%), 2023 (January to June, 26.49%), and 2020 (June to December, 10.45%). In total, sex was reported in 122 (45.52%) studies, and 4 (1.49%) studies explained the limitation of sex. Gender was reported in 35 (13.06%) studies, and 7 (2.61%) studies explained the limitation of gender. Globally, a total of 96 (35.82%) studies reported race, ethnicity, or ancestry data in the study, and a total of 43 (16.04%) studies explained the limitation of race, ethnicity, or ancestry (Table 1).

In 2020, 46.43% of studies reported either race, ethnicity, or ancestry, and the percentages were lower in subsequent years: 2021 (24.10%), 2022 (44.19%), and 2023 (35.21%). Sex and gender were reported for 53.57% and 10.71% of studies in 2020, 39.76% and 6.02% in 2021, 50.00% and 17.44% in 2022, and 43.66% and 16.90% in 2023, respectively. There were significant differences between study years in the reporting of race, ethnicity, or ancestry (*p* = 0.028, Fisher’s exact test), but pairwise comparisons were not statistically significant (Figure 2, Table 2).

### 3.2. Results from NIH-Funded Studies

A total of 90 AI studies in the overall analysis reported NIH funding in the publication. Among these studies, the top three journals in which these studies were published included American Journal of Ophthalmology (n = 37, 41.1%), Ophthalmology (n = 19, 21.1%), and British Journal of Ophthalmology (n = 13, 14.4%). The majority of articles were published in 2021 (n = 43, 47.8%). Of the NIH-funded studies, 44 (48.9%) reported sex, 14 (15.6%) reported gender, 41 (45.6%) reported race, and 31 (34.4%) reported ethnicity. In these studies, one (1.1%), two (2.2%), four (4.4%), and five (5.6%) reported limitations of sex, gender, race, or ethnicity, respectively. Of the studies that reported sex, gender, race, and ethnicity, 29 (65.9%), 2 (14.3%), 10 (24.4%), and 1 (3.2%) followed NIH reporting guidelines, respectively. In addition, in those studies that did report sex, gender, race, and ethnicity, 12 (27.3%), 0 (0.0%), 7 (17.1%), and 1 (3.2%) reported how these demographics were assessed. (Table 3).

Reporting of race in NIH-funded studies increased substantively from 11.1% in 2020 to 72.1% in 2021. This was followed by a decline in 2022 to 18.2% and a modest increase to 31.3% in 2023. Thus, reporting peaked in 2021 rather than showing a consistent upward trend over time. Similar patterns were observed for ethnicity, with reporting rising from 11.1% in 2020 to 58.1% in 2021. Ethnicity reporting in 2022 declined to 9.1%, and in 2023 increased slightly to 18.8%. Sex was reported in 55.6% of studies in 2020, 48.8% in 2021, 40.9% in 2022, and 56.3% in 2023. Gender was not reported by any studies in 2020, but was reported by 27.9% in 2021 and 9.1% in 2022, and was not reported by any studies in 2023. Differences in the percentage of studies reporting gender (*p* = 0.020), race (*p* < 0.001), and ethnicity (*p* < 0.001) between the study years were observed. Statistically significant pairwise comparisons were observed for both race and ethnicity. Specifically, for race, differences between years 2020 and 2021 (*p* = 0.001), 2021 and 2022 (*p* < 0.0001), and 2021 and 2023 (*p* = 0.007) were observed. After applying Holm adjustments, these differences remained significant (*p* = 0.006, *p* < 0.001, and *p* = 0.027, respectively). In addition, for ethnicity, statistically significant pairwise comparisons were observed between years 2020 and 2021 (*p* = 0.024), 2021 and 2022 (*p* < 0.001), and 2021 and 2023 (*p* = 0.009). Even after applying Holm adjustments, pairwise comparisons between years 2021 and 2022 (*p* = 0.001) and 2021 and 2023 (*p* = 0.044) remained statistically significant. No pairwise comparisons were statistically significant for sex and gender (Table 4, Figure 3).

### 3.3. United States-Based Studies

A total of 74 AI studies were identified between June 2020 and June 2023 in the sub-analysis of US-based AI studies. One study in the full analysis was not considered, as the location of the study could not be determined. Among the 74 studies, the top three journals were Ophthalmology (*n =* 18, 24.3%), British Journal of Ophthalmology (*n* = 14, 18.9%), and Ophthalmology (*n =* 13, 17.6%). The full list of journals and numbers of US-based studies is listed in Table 5. Articles were published from 2020 to 2023; most were published in 2022 (33.8%), followed by 2023 (25.7%), 2021 (21.6%), and 2020 (18.9%). (Table 5)

Of the US-based studies, 33 (44.6%) reported sex, and the remaining 41 (55.4%) did not. Of those that reported sex, 15 (45.5%) studies followed NIH guidelines for reporting sex, and 13 (39.4%) reported how sex was assessed. There were two (2.7%) studies that explained the limitation of sex in the study. In the US sub-analysis, four (4.5%) studies reported gender. Of the studies that reported gender, none of them followed NIH reporting guidelines or reported how gender was assessed in their study. There were three (4.1%) studies that explained the limitation of gender (Table 5).

There were 21 (48.31%) studies that reported race in the study, and of those studies, 11 (52.4%) studies followed NIH guidelines for reporting race and 10 (47.6%) of those reported how race was assessed. There were five (6.8%) studies that explained the limitation of race in the study. In total, 10 (13.5%) reported ethnicity in the study. Of these studies, one (10%) followed NIH Guidelines for reporting ethnicity, and one reported how ethnicity was assessed (10%). There were four (5.4%) studies that explained the limitation of ethnicity in the study (Table 5).

Race reporting increased sharply from 14.3% in 2020 to 43.8% in 2021, then declined to 20.0% in 2022 and rose modestly to 36.8% in 2023. Thus, similar to the NIH sub-analysis, reporting peaked in 2021 rather than showing a consistent upward trend over time. Ethnicity was reported in 14.3% of studies in 2020, 6.3% in 2021, 4.00% in 2022, and 31.6% in 2023. Gender was reported in 0% of studies in 2020, 6.3% in 2021, 8.0% in 2022, and 5.4% in 2023. Sex was reported in 50.00% of studies in 2020, 50.0% in 2021, 28.0% in 2022, and 57.9% in 2023. Differences in the percentage of studies reporting ethnicity between the study years were observed (*p* = 0.051). A lower percentage of studies reported ethnicity in 2022 compared with 2023 (4.0% vs 31.6%, *p* = 0.032). This was the only analysis that showed a Holm-adjusted statistically significant lower percentage of studies reporting ethnicity for 2022 compared to 2023. No pairwise comparisons were statistically significant for sex, gender, or race reporting (Table 6, Figure 4).

## 4. Discussion

In this investigation of AI studies published from 2020 to 2023 in highly cited ophthalmology journals, a little over half reported on the gender or sex (58.6%) of their population cohorts, while only about one-third reported information on race, ethnicity, or ancestry of their study cohorts (35.8%). Furthermore, over 90% of AI studies did not discuss the limitations of gender or sex reporting in the study, and over 80% did not discuss the limitations of race, ethnicity, or ancestry reporting in the study. In the sub-analysis of NIH-funded studies, reporting of both race and ethnicity was significantly higher in 2021 than in 2022 and 2023, even after Holm adjustments for multiple comparisons. The only increase observed was for ethnicity in studies that included US data, where ethnicity was reported significantly less often in 2022 compared to 2023. This study adds to the literature by systematically quantifying these demographic reporting gaps in highly cited ophthalmology journals and evaluating whether authors acknowledge their implications as study limitations. These findings provide a field-specific benchmark that can guide reporting standards and improve the transparency, generalizability, and equitable deployment of ophthalmic AI. Since knowing the characteristics of the populations in which AI algorithms were developed is crucial to understanding the potential impact of deploying those AI algorithms, there is room to improve reporting on race, gender, sex, and ethnicity within the field of AI in ophthalmology.

Ophthalmology is a recognized leader in the field of AI for healthcare. The first autonomous AI device cleared by the FDA was the IDx-DR for detecting diabetic retinopathy [23]. As such, we have a particular responsibility as leaders in the field to ensure the responsible use of AI and promote fair and equitable AI. Bondok et al. found in AI studies in retinal diseases that there was an underrepresentation of sociodemographic groups, as well as insufficient reporting of ethnic and racial data [24]. The authors noted that improved reporting is needed to ensure that AI tools can perform well across multiple populations with retinal conditions. The potential harms of bias in algorithms have also been amply demonstrated in other fields. Obermeyer et al.’s paper in Science highlights how bias in an algorithm can exacerbate health disparities [25]. The authors investigated a widely used algorithm that had been applied to around 200 million individuals yearly in the United States and targeted individuals who would benefit from entering high-risk programs intended for individuals with high disease risk and health consequences. The authors identified a bias in the algorithm that was confirmed by the algorithm manufacturer, who ran an independent analysis. If corrected, the number of Black individuals who would receive additional care would increase from 17.7% to 46.5% [25]. This powerful study highlights that detailed analysis of the impact of algorithms on different subpopulations of patients is only possible if demographic information is reported in the first place. Potential users/consumers of various algorithms cannot begin to know the potential impacts of these algorithms on their own patient populations without transparent reporting on the population on which the algorithms were trained. A necessary prerequisite to fair AI is understanding how AI algorithms are created and which populations are included in their training. Thus, demographic reporting is crucial here. Demographic underreporting may have specific impacts in ophthalmology that can translate into direct clinical consequences. For example, Chen et al. observed very specific phenotypic differences in diabetic retinopathy fundus imaging between ethnicities despite similar age and glycemic control [26]. Our study shows that much more progress is needed before our field achieves 100% reporting of sex, race, and ethnicity in AI studies. Adoption of standards for AI publications that require reporting of demographic information might promote greater transparency in reporting in the future. Clear reporting can also aid in moving the field to focus on data gathering from diverse populations. Ophthalmology journals could implement data reporting guidelines for AI studies to help facilitate this process. Work in this field is underway in ophthalmology and in general healthcare [27,28].

Interestingly, in this study, in the United States, there was no improvement in reporting of gender, race, and ethnicity following 2021. This study also highlights that even in NIH-funded studies, there was an increase in race and ethnicity reporting in 2021, but that reporting in 2021 was statistically significantly higher than in subsequent years. In 2020, the COVID pandemic was observed to exacerbate healthcare inequalities [29,30,31,32,33]. Major journals, including *NEJM Catalyst* and *Bulletin of the World Health Organization*, published articles regarding health equity to reduce these inequalities for disadvantaged communities, potentially leading to higher reporting in 2021 [30,33]. However, this improvement was not sustained, suggesting that heightened attention to health equity during the pandemic may not have produced a durable change in reporting practices. By documenting this temporal pattern specifically within ophthalmic AI research, the present study demonstrates that increased awareness of healthcare disparities alone may be insufficient to ensure consistent demographic transparency. Although the observational design precludes attributing the 2021 increase directly to pandemic-related attention, our findings establish a field-specific benchmark and underscore the need for standardized journal, conference, and reporting guideline requirements. Initiatives, such as mandatory demographic reporting, justification of omitted variables, and explicit discussion of limitations related to population representativeness, remain necessary to improve the transparency, generalizability, and equitable clinical application of ophthalmic AI.

This study has various merits and limitations. To our knowledge, this is the first study that characterizes and analyzes the reporting of race, gender, sex, and ethnicity in ophthalmology AI studies from multiple journals at this scale. In developing equitable AI, our community has room to improve. This study has limitations. A notable limitation is that the data did not include the full years of 2020 or 2023. This was because in 2020, the calls to action did not occur until health inequity was highlighted by the COVID-19 pandemic and the George Floyd protests. The literature search for this study began in 2023; thus, we did not have the full year of data. Although the full years of 2021 and 2022 were collected, collection of the other half-years may provide deeper insights into the reporting that occurred during those years. Furthermore, the publication year cannot establish when reporting decisions were made or support causal attribution of the 2021 increase. The sample size was relatively small, so some results may not have had sufficient statistical power to detect differences. Future research can focus on extending the publication period to increase the sample size and statistical power. The larger sample size can also be used to compare data before and after the updated guidelines. This study also focused on the most cited ophthalmology journals. The issues raised could be more or less present in other journals. In addition, our classification relied on reported terminology; we could not determine what the labels measured; therefore, findings reflect adherence to reporting recommendations, not measurement validity. Furthermore, this study focused on reporting transparency rather than whether populations were underrepresented, whether the models were biased, or whether the algorithms were equitable or generalizable. However, transparency is a prerequisite for evaluating these issues. Finally, we only searched PubMed because we limited our search to a specific set of journals; however, this approach may have limited our ability to identify all relevant articles, particularly those not indexed in PubMed.

The field of ophthalmology is a leader in improving future patient care with AI. Ophthalmology AI studies of the future must ensure that these technologies optimize health for large populations rather than exacerbate health disparities [24]. Future policymakers or journal editorial boards in ophthalmology may consider implementing consistent reporting guidelines on race, gender, sex, and ethnicity in AI studies to help optimize outcomes (Online Supplemental Table S1).

## Figures and Tables

**Figure 1:**
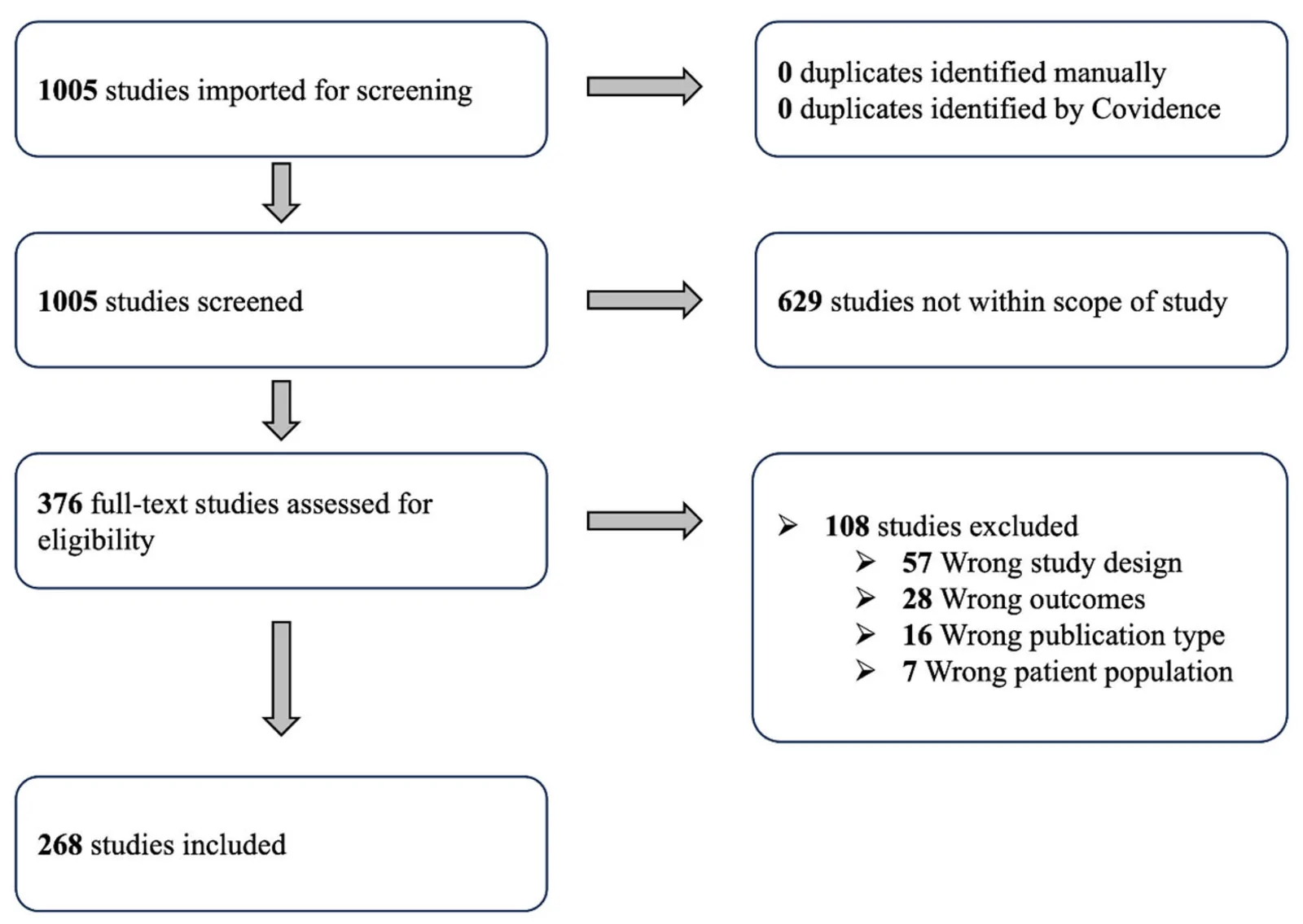
Flowchart describing the inclusion of studies screened for this study. Wrong study designs included studies that were not AI studies. Wrong outcomes indicated studies that were not about ocular disease or vision-specific. Wrong publication type indicated case studies, reviews, and abstracts.

**Figure 2:**
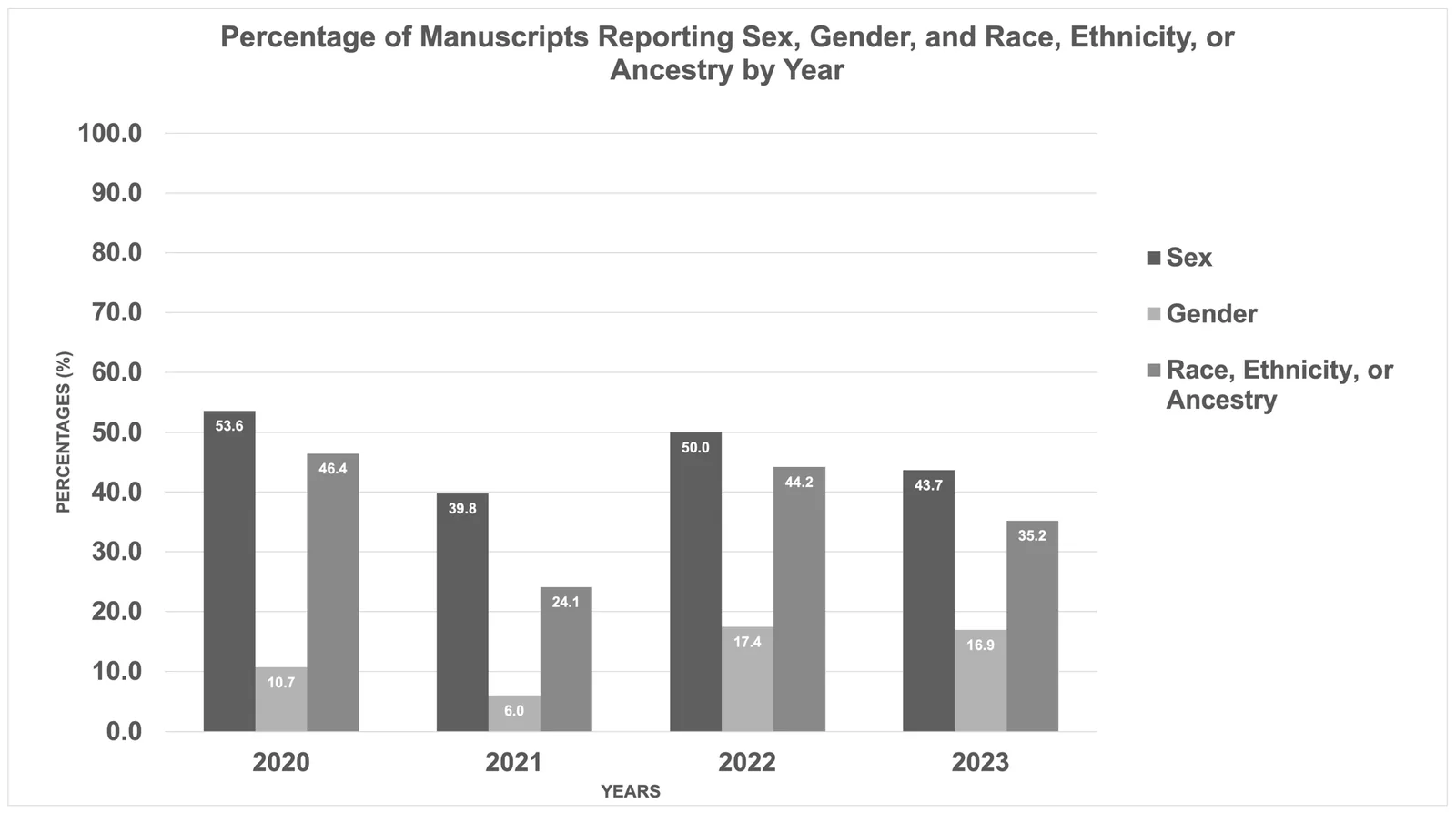
Percentage of Manuscripts Reporting Sex, Gender, Race, and Ethnicity by Year.

**Figure 3:**
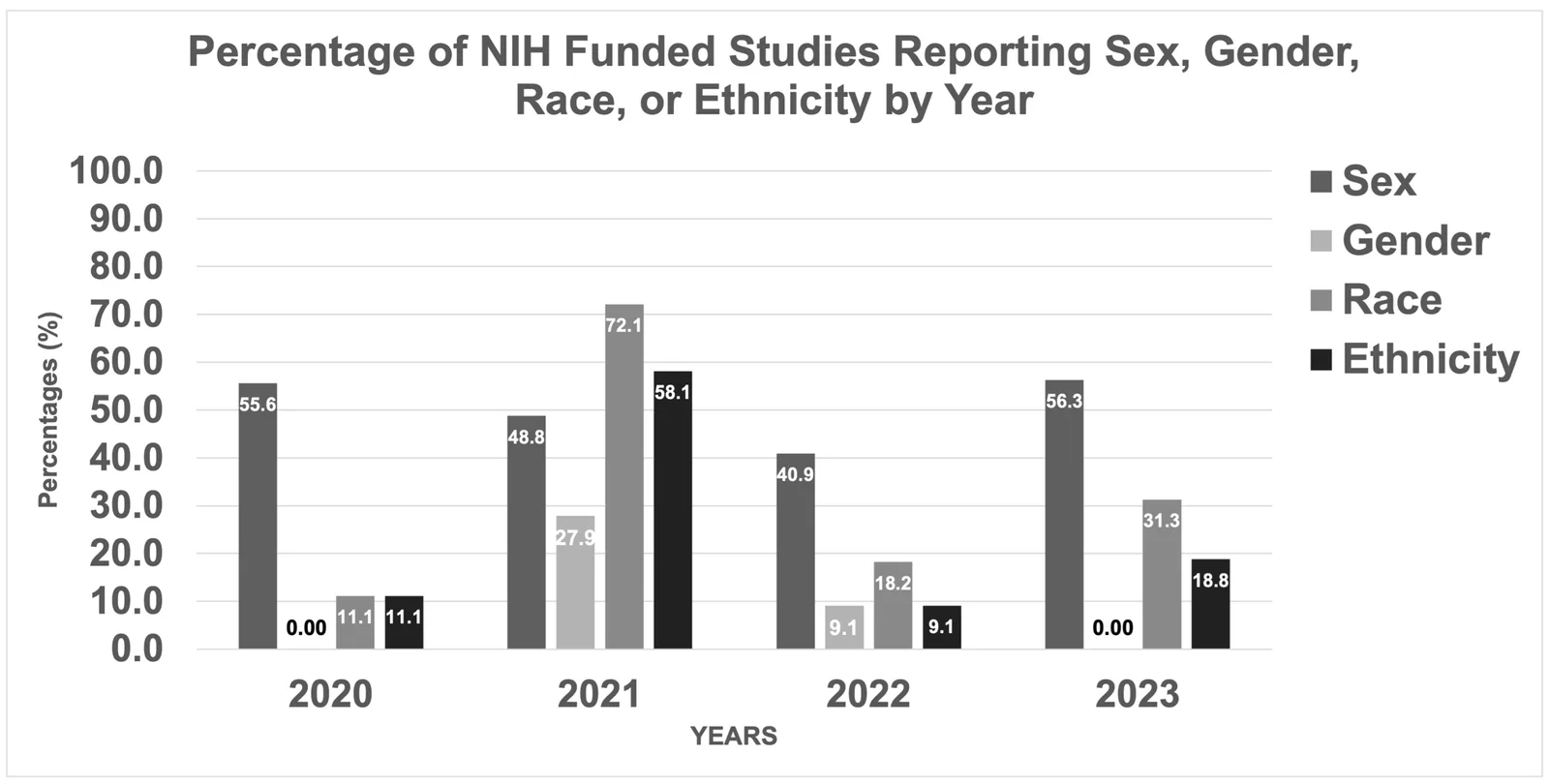
Percentage of NIH-funded studies reporting Sex, Gender, Race, or Ethnicity.

**Figure 4:**
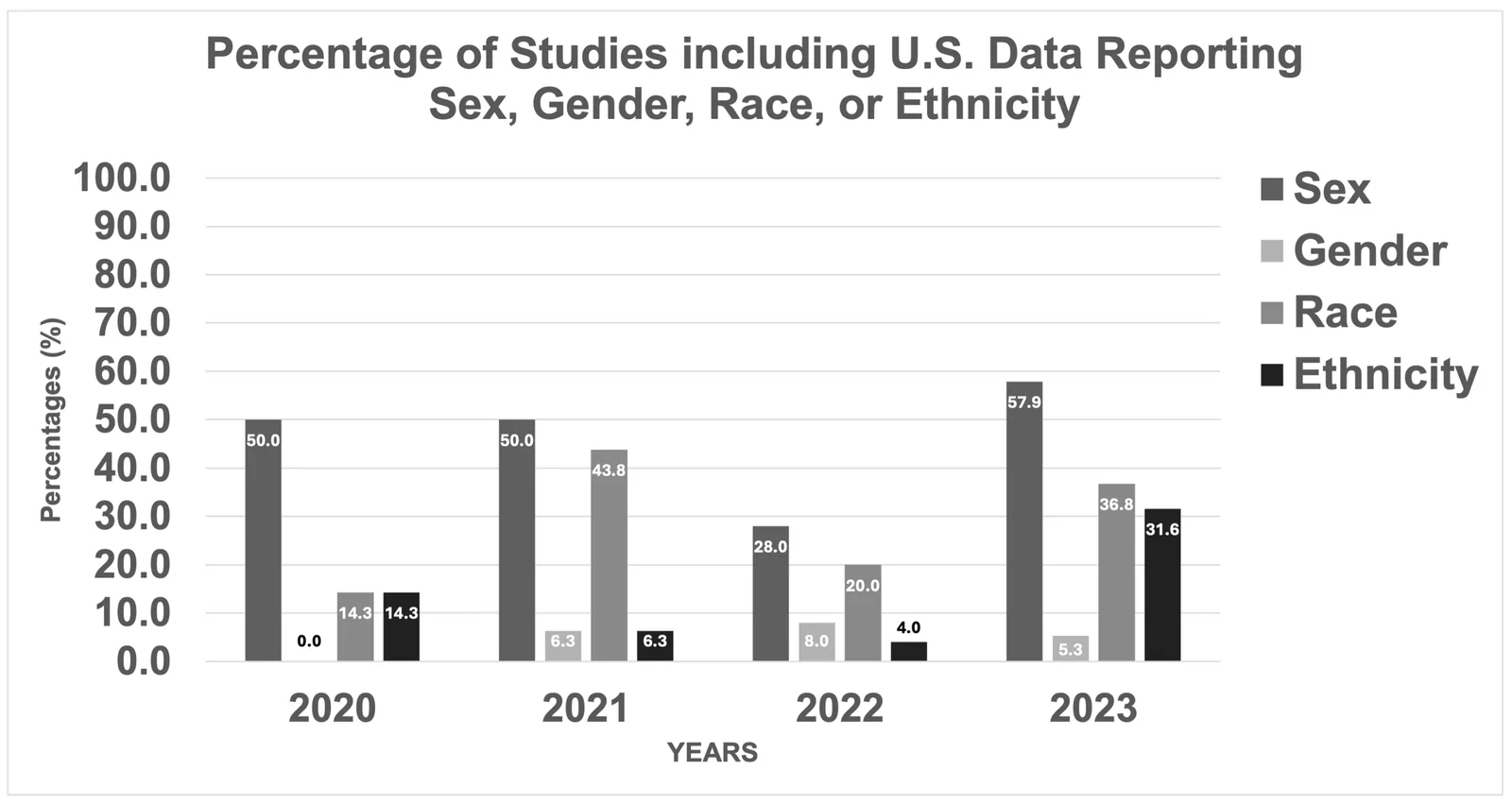
Percentage of Studies including U.S. Data Reporting Sex, Gender, Race, or Ethnicity.

**Table 1:** Characteristics of Included Studies (*n* = 268).

Publication Characteristic	Total Studies (*n* = 268)
Journal	
Acta Ophthalmology	14 (5.22%)
American Journal of Ophthalmology	60 (22.39%)
Ophthalmology	26 (9.70%)
British Journal of Ophthalmology	63 (23.51%)
Cornea	9 (3.36%)
Experimental Eye Research	1 (0.37%)
Graefe’s Archive for Clinical and Experimental Ophthalmology	22 (8.21%)
Investigative Ophthalmology and Visual Science	6 (2.24%)
Journal of Cataract and Refractive Surgery	5 (1.87%)
JAMA Ophthalmology	14 (5.22%)
Journal of Vision	2 (0.75%)
Retina	23 (8.58%)
The Royal College of Ophthalmologists (Eye)	23 (8.58%)
Year	
2020	10.45%
2021	30.97%
2022	32.09%
2023	26.49%
Reported Sex in the study	
Yes	122 (45.52%)
No	146 (54.48%)
Limitation of Sex explained in the study	
Yes	4 (1.49%)
No	264 (98.51%)
Reported Gender in the study	
Yes	35 (13.06%)
No	233 (86.94%)
Limitation of Gender explained in the study	
Yes	7 (2.61%)
No	261 (97.39%)
Reported Race, Ethnicity, or Ancestry in the study	
Yes	96 (35.82%)
No	172 (64.18%)
Limitation of Race, Ethnicity, or Ancestry explained in the study	
Yes	43 (16.04%)
No	225 (83.96%)

**Table 2:** Publication Year and Demographic Reporting Status in U.S. and International AI Studies.

Characteristic	Years					*p*-Value *
	2020 (*n =* 28)	2021 (*n =* 83)	2022 (*n =* 86)	2023 (*n =* 71)	Total (*n =* 268)	
Sex Reported—Yes	15 (53.6%)	33 (39.8%)	43 (50.0%)	31 (43.7%)	122 (45.5%)	0.454
Sex Reported—No	13 (46.4%)	50 (60.2%)	43 (50.0%)	40 (56.3%)	146 (54.5%)	
Gender Reported—Yes	3 (10.7%)	5 (6.0%)	15 (17.4%)	12 (16.9%)	35 (13.1%)	0.087
Gender Reported—No	25 (89.3%)	78 (94.0%)	71 (82.6%)	59 (83.1%)	233 (86.9%)	
Race, Ethnicity, or Ancestry Reported—Yes	13 (46.4%)	20 (24.1%)	38 (44.2%)	25 (35.2%)	96 (35.8%)	0.028 **
Race, Ethnicity, or Ancestry Reported—No	15 (53.6%)	63 (75.9%)	48 (55.8%)	46 (64.8%)	172 (64.2%)	

* Fisher’s Exact test *p*-value. ** Fisher’s exact test; Holm’s pairwise comparisons were not statistically significant.

**Table 3:** Characteristics of NIH-funded studies in the sub-analysis (*n =* 90).

Publication Characteristics	Total Studies (*n =* 90)
Journal	
American Journal of Ophthalmology	37 (41.1%)
Ophthalmology	19 (21.1%)
British Journal of Ophthalmology	13 (14.4%)
Cornea	4 (4.4%)
Investigative Ophthalmology and Visual Science	3 (3.3%)
JAMA Ophthalmology	6 (6.7%)
Journal of Vision	1 (1.1%)
Retina	4 (4.4%)
The Royal College of Ophthalmologists (Eye)	3 (3.3%)
Year	
2020	9 (10.0%)
2021	43 (47.8%)
2022	22 (24.4%)
2023	16 (17.8%)
Reported Sex in the study	
Yes	44 (48.9%)
No	46 (51.1%)
Followed NIH Guidelines for reporting Sex	
Yes	29 (65.9%)
No	15 (34.1%)
Not Applicable	46
Was limitation of Sex explained in the study	
Yes	1 (1.1%)
No	89 (98.9%)
Reported how Sex was assessed in the study	
Yes	12 (27.3%)
No	32 (72.7%)
Not Applicable	46
Reported Gender in the study	
Yes	14 (15.6%)
No	76 (84.4%)
Followed NIH Guidelines for reporting Gender	
Yes	2 (14.3%)
No	12 (85.7%)
Not Applicable	76
Was limitation of Gender explained in the study	
Yes	2 (2.2%)
No	88 (97.8%)
Reported how Gender was assessed in the study	
Yes	0 (0.0%)
No	14 (100.0%)
Not Applicable	76
Reported Race in the study	
Yes	41 (45.6%)
No	49 (54.4%)
Followed NIH Guidelines for reporting Race	
Yes	10 (24.4%)
No	31 (75.6%)
Not Applicable	49
Was limitation of Race explained in the study	
Yes	4 (4.4%)
No	86 (95.6%)
Reported how Race was assessed in the study	
Yes	7 (17.1%)
No	34 (82.9%)
Not Applicable	49
Reported Ethnicity in the study	
Yes	31 (34.4%)
No	59 (65.6%)
Followed NIH Guidelines for reporting Ethnicity	
Yes	1 (3.2%)
No	30 (96.8%)
Not Applicable	59
Was limitation of Ethnicity explained in the study	
Yes	5 (5.6%)
No	85 (94.4%)
Reported how Ethnicity was assessed in the study	
Yes	1 (3.2%)
No	30 (96.8%)
Not Applicable	59

**Table 4:** Publication Year and Demographic Reporting Status in Studies with NIH Funding.

Characteristic	Years					*p*-Value *
	2020	2021	2022	2023	Total	
Sex Reported—Yes	5 (55.6%)	21 (48.8%)	9 (40.9%)	9 (56.3%)	44 (48.9%)	0.819
Sex Reported—No	4 (44.4%)	22 (51.2%)	13 (59.1%)	7 (43.8%)	46 (51.1%)	
Gender Reported—Yes	0 (0.0%)	12 (27.9%)	2 (9.1%)	0 (0.0%)	14 (15.6%)	0.020 ^c,^**
Gender Reported—No	9 (100.0%)	31 (72.1%)	20 (90.9%)	16 (100.0%)	76 (84.4%)	
Race Reported—Yes	1 (11.1%)	31 (72.1%)	4 (18.2%)	5 (31.3%)	41 (45.6%)	<0.001 ^a,b,c,d,e,f^
Race Reported—No	8 (88.9%)	12 (27.9%)	18 (81.8%)	11 (68.8%)	49 (54.4%)	
Ethnicity Reported—Yes	1 (11.1%)	25 (58.1%)	2 (9.1%)	3 (18.8%)	31 (34.4%)	<0.001 ^a,b,c,e,f^
Ethnicity Reported—No	8 (88.9%)	18 (41.9%)	20 (90.9%)	13 (81.3%)	59 (65.6%)	

* Fisher’s Exact test *p*-value. ** Fisher’s exact test; Holm’s pairwise comparisons were not statistically significant for any groups. ^a^: Pairwise comparison years 2020 and 2021 <0.05. ^b^: Pairwise comparison between years 2021 and 2022 <0.05. ^c^: Pairwise comparison between years 2021 and 2023 <0.05. ^d^: Pairwise comparison between years 2020 and 2021 <0.05 with Holm adjustment. ^e^: Pairwise comparison between years 2021 and 2022 <0.05 with Holm adjustment. ^f^: Pairwise comparison between years 2021 and 2023 <0.05 with Holm adjustment.

**Table 5:** Overview of the studies including data from the U.S. (*n =* 74).

Publication Characteristics	Total Studies (*n =* 74)
Journal	
American Journal of Ophthalmology	13 (17.6%)
Ophthalmology	18 (24.3%)
British Journal of Ophthalmology	14 (18.9%)
Cornea	4 (5.4%)
Graefe’s Archive of Clinical and Experimental Ophthalmology	3 (4.1%)
Investigative Ophthalmology and Visual Science	1 (1.4%)
JAMA Ophthalmology	9 (12.2%)
Journal of Vision	1 (1.4%)
Retina	7 (9.5%)
The Royal College of Ophthalmologists (Eye)	2 (2.7%)
Year	
2020	14 (18.9%)
2021	16 (21.6%)
2022	25 (33.8%)
2023	19 (25.7%)
Reported Sex in the study	
Yes	33 (44.6%)
No	41 (55.4%)
Followed NIH Guidelines for reporting Sex	
Yes	15 (45.5%)
No	18 (54.5%)
Not Applicable	41
Was limitation of Sex explained in the study	
Yes	2 (2.7%)
No	72 (97.3%)
Reported how Sex was assessed in the study	
Yes	13 (39.4%)
No	20 (60.6%)
Not Applicable	41
Reported Gender in the study	
Yes	4 (5.4%)
No	70 (94.6%)
Followed NIH Guidelines for reporting Gender	
Yes	0 (0.0%)
No	4 (100.0%)
Not Applicable	70
Was limitation of Gender explained in the study	
Yes	3 (4.1%)
No	71 (95.9%)
Reported how Gender was assessed in the study	
Yes	0 (0.0%)
No	4 (100.0%)
Not Applicable	70
Reported Race in the study	
Yes	21 (28.4%)
No	53 (71.6%)
Followed NIH Guidelines for reporting Race	
Yes	11 (52.4%)
No	10 (47.6%)
Not Applicable	53
Was limitation of Race explained in the study	
Yes	5 (6.8%)
No	69 (93.2%)
Reported how Race was assessed in the study	
Yes	10 (47.6%)
No	11 (52.4%)
Not Applicable	53
Reported Ethnicity in the study	
Yes	10 (13.5%)
No	64 (86.5%)
Followed NIH Guidelines for reporting Ethnicity	
Yes	1 (10.0%)
No	9 (90.0%)
Not Applicable	64
Was limitation of Ethnicity explained in the study	
Yes	4 (5.4%)
No	70 (94.6%)
Reported how Ethnicity was assessed in the study	
Yes	1 (10.0%)
No	9 (90.0%)
Not Applicable	64

**Table 6:** Publication Year and Demographic Reporting Status in the U.S. AI Studies.

Characteristic	Years				*p*-Value *
	2020	2021	2022	2023	
Sex Reported—Yes	7 (50.0%)	8 (50.0%)	7 (28.0%)	11 (57.9%)	0.213
Sex Reported—No	7 (50.0%)	8 (50.0%)	18 (72.0%)	8 (42.1%)	
Gender Reported—Yes	0 (0.0%)	1 (6.3%)	2 (8.0%)	1 (5.3%)	0.908
Gender Reported—No	14 (100.0%)	15 (93.8%)	23 (92.0%)	18 (94.7%)	
Race Reported—Yes	2 (14.3%)	7 (43.8%)	5 (20.0%)	7 (36.8%)	0.205
Race Reported—No	12 (85.7%)	9 (56.3%)	20 (80.0%)	12 (63.2%)	
Ethnicity Reported—Yes	2 (14.3%)	1 (6.3%)	1 (4.0%)	6 (31.6%)	0.051 ^a**^
Ethnicity Reported—No	12 (85.7%)	15 (93.8%)	24 (96.0%)	13 (68.4%)	

* Fisher’s Exact test *p*-value. ** Fisher’s exact test; Holm’s pairwise comparisons were not statistically significant. ^a^: Pairwise comparison years 2022 and 2023 <0.05.

## Data Availability

Data available upon reasonable request to the corresponding author.

## Supplementary Materials

The following supporting information can be downloaded at https://www.mdpi.com/article/10.3390/vision10040072/s1, Figure S1: Literature Search; Table S1: Demographic Definitions.

## Abbreviations

The following abbreviations are used in this manuscript:

AI	Artificial intelligence
NIH	National Institutes of Health
