# Supplementary material for: Multi-Year Analysis of Demographic Reporting in Ophthalmology Artificial Intelligence Studies
Source: Vision (Basel). 2026 Sep 25;10(4):72. doi: 10.3390/vision10040072 (PMC13709598; doi:10.3390/vision10040072)
Supplement: Supplementary file 1 [file vision-10-00072-s001.zip › Figure S1 Literature Search.pdf]

Figure S1: Review Search Terms

The PubMed database was selected for utilization in this review. The search terms in Point 1 and the journals in Point 2 were inputted into the criterion and searched from the years 2020 to 2023.

1. "Artificial Intelligence"[Mesh] OR NLP OR LLM OR "Artificial intelligence" OR “artificial general intelligence” OR “artificial social intelligence” OR "Expert Systems" OR "Fuzzy Logic" OR "Knowledge Base" OR "Machine Learning" OR "Natural Language Processing" OR “Natural language understanding” OR "computer neural networks" OR "Deep Learning" OR "Sentiment Analys*" OR "artificial neural network" OR “artificial narrow intelligence” OR “augmented intelligence” OR “intelligence augment” OR “optical character recognition” OR “cognitive comput” OR “recurrent neural network” OR “deep neural network” OR "naive bayes" OR "support vector" OR "random forest" OR "large language model" OR "logistic model"[tiab:~2] OR "logistic models"[tiab:~2] OR "logistic regression"[tiab:~2] OR "Heuristic" OR "knowledge engineer"[tiab:~2] OR "machine intelligence"[tiab:~2] OR "google bard" OR "ChatGPT" OR "GPT-3" OR "GPT-3.5" OR "GPT-4" OR “generative AI” OR “chatbot” OR “conversational AI” OR “robotic process automation” OR “AI healthcare” OR “AI health care” OR “alphago” OR “dental AI” OR “medical AI” OR “AI application” OR “AI apps” OR “AI app” OR “deep mesh” OR “Open AI” OR “GPT” OR “generative pre-trained transformer” OR “generative pretrained transformer” OR "OpenAI"
753256
2. (top 15 from SCIE, sorted by total citations not JIF)
"Invest Ophthalmol Vis Sci"[Journal] OR "Ophthalmology"[Journal] OR "Am J Ophthalmol"[Journal] OR "Br J Ophthalmol"[Journal] OR "Exp Eye Res"[Journal] OR "Eye (Lond)"[Journal] OR "Vision Res"[Journal] OR "J Cataract Refract Surg"[Journal] OR "Retina"[Journal] OR "Cornea"[Journal] OR "Acta Ophthalmol"[Journal] OR "Graefes Arch Clin Exp Ophthalmol"[Journal] OR "Prog Retin Eye Res"[Journal] OR "JAMA Ophthalmol"[Journal] OR "J Vis"[Journal]
1 AND 2 3196

Filters applied: from 2020/6/1 - 2023/6/30 1005 results 11/1/23
