# Supplementary material for: Multi-Year Analysis of Demographic Reporting in Ophthalmology Artificial Intelligence Studies
Source: Vision (Basel). 2026 Sep 25;10(4):72. doi: 10.3390/vision10040072 (PMC13709598; doi:10.3390/vision10040072)
Supplement: Supplementary file 1 [file vision-10-00072-s001.zip › Table S1 Demographic Definitions.pdf]

Table S1: Demographic Definitions

Demographic Variable	Definitions*	Examples
Race[19-20]	a social construct designed to categorize people and divides human populations into groups typically based on physical appearance, social factors, and cultural backgrounds.	American Indian or Alaska Native<br>Asian<br>Black or African American<br>Native Hawaiian or Other Pacific Islander<br>White or Caucasian
Ethnicity[21]	the social and cultural characteristics, backgrounds, or experiences shared by a group of people, including language, religion, beliefs, values, and behaviors that are often passed down from one generation to the next.	Hispanic<br>Non-Hispanic
Sex[17]	a multidimensional biological construct based on anatomy, physiology, genetics, and hormones.	Female<br>Male<br>Intersex
Gender[18]	a multidimensional construct that encompasses gender identity and expression, as well as social and cultural expectations about status, characteristics, and behavior as they are associated with certain sex traits.	Woman<br>Man<br>Transgender<br>Non-binary/non-conforming
